# The mitochondrial genome of the diploid oat *Avena longiglumis*

**DOI:** 10.1186/s12870-023-04217-8

**Published:** 2023-04-26

**Authors:** Qing Liu, Hongyu Yuan, Jiaxin Xu, Dongli Cui, Gui Xiong, Trude Schwarzacher, John Seymour Heslop-Harrison

**Affiliations:** 1 Key Laboratory of Plant Resources Conservation and Sustainable Utilization / Guangdong Provincial Key Laboratory of Applied Botany, South China Botanical Garden, Chinese Academy of Sciences, Guangzhou, 510650 China; 2 South China National Botanical Garden, Guangzhou, 510650 China; 3 Center for Conservation Biology, Core Botanical Gardens, Chinese Academy of Sciences, Guangzhou, 510650 China; 4 University of Chinese Academy of Sciences, Beijing, 100049 China; 5 College of Plant Protection, South China Agricultural University, Guangzhou, 510642 China; 6 Department of Genetics and Genome Biology, Institute for Environmental Futures, University of Leicester, Leicester, LE1 7RH UK

**Keywords:** *Avena longiglumis*, Chloroplast, Genome recombination, Mitochondrial genome assembly, Mitogenome, Mitochondrial nuclear DNAs (MTNUs), Mitochondrial plastid DNAs (MTPTs), Organelle, Plants, RNA editing

## Abstract

**Background:**

*Avena longiglumis* Durieu (2*n* = 2*x* = 14) is a wild relative of cultivated oat (*Avena sativa*, 2*n* = 6*x* = 42) with good agronomic and nutritional traits. The plant mitochondrial genome has a complex organization and carries genetic traits of value in exploiting genetic resources, not least male sterility alleles used to generate F_1_ hybrid seeds. Therefore, we aim to complement the chromosomal-level nuclear and chloroplast genome assemblies of *A. longiglumis* with the complete assembly of the mitochondrial genome (mitogenome) based on Illumina and ONT long reads, comparing its structure with Poaceae species.

**Results:**

The complete mitochondrial genome of *A. longiglumis* can be represented by one master circular genome being 548,445 bp long with a GC content of 44.05%. It can be represented by linear or circular DNA molecules (isoforms or contigs), with multiple alternative configurations mediated by long (4,100–31,235 bp) and medium (144–792 bp) size repeats. Thirty-five unique protein-coding genes, three unique rRNA genes, and 11 unique tRNA genes are identified. The mitogenome is rich in duplications (up to 233 kb long) and multiple tandem or simple sequence repeats, together accounting for more than 42.5% of the total length. We identify homologous sequences between the mitochondrial, plastid and nuclear genomes, including the exchange of eight plastid-derived tRNA genes, and nuclear-derived retroelement fragments. At least 85% of the mitogenome is duplicated in the *A. longiglumis* nuclear genome. We identify 269 RNA editing sites in mitochondrial protein-coding genes including stop codons truncating *ccmFC* transcripts.

**Conclusions:**

Comparative analysis with Poaceae species reveals the dynamic and ongoing evolutionary changes in mitochondrial genome structure and gene content. The complete mitochondrial genome of *A. longiglumis* completes the last link of the oat reference genome and lays the foundation for oat breeding and exploiting the biodiversity in the genus.

**Supplementary Information:**

The online version contains supplementary material available at 10.1186/s12870-023-04217-8.

## Background

As a widely grown temperate food crop, oats have long been favored by consumers because their grains are rich in nutrients such as protein, fat, vitamin B1, and β-glucan, as well as being a source of carbohydrates with low glycaemic index, dietary soluble fiber, different phenolic compounds, and minerals [1–4]. Oat is used as a high-quality feed for livestock with significant economic value [5].

Common oat (*Avena sativa* L.; abbreviated here ASA, 2*n* = 6*x* = 42, genome composition AACCDD; Poaceae family) has a complex evolutionary origin, involving the separation of species from a common ancestor and then hybridization of an ancestral diploid and an ancestral tetraploid species, with further possible introgression from other ancestors [6]. *Avena longiglumis* Durieu (ALO, 2*n* = 2*x* = 14, AA) is annual, grows primarily in the Mediterranean biome, and is considered a wild diploid ancestor to the common oat [7, 8]. As a crop wild relative, *A. longiglumis* has various agronomic traits, including stress resistance and nutritional traits, which are valuable to oat breeders.

Our extensive comparative analysis of the plastid (chloroplast) genomes of 13 *Avena* accessions identified the presence of rearrangement events in the plastomes [9], complementing other assemblies of oat plastomes [10, 11]. Due to the large genome size, presence of many repetitive DNA sequences, and complex ploidy in the oat nuclear genome, genomic studies lagged behind other gramineous crops, such as rice, maize and barley. It was not until 2022 that Peng et al. [12], Kamal et al. [13] and Liu et al. [14] published the reference nuclear genomes of cultivated oat and their diploid and tetraploid ancestors. Among them, the nuclear genome size of *A*. *longiglumis* was about 3.8 Gb.

In animals, mitochondrial genomes are mostly about 16 to 17 kb long, as a single circular assembly molecule with species-specific variation, making them extremely useful for phylogenetics [15, 16]. In contrast, angiosperm mitogenomes are much larger (typically 400 kb), with substantial variation in size, architecture, and extensive rearrangements [17] showing evidence of frequent sequence migration to plastid and nuclear genomes. Within species, there are with multiple complex configurations and this dynamic mitochondrial genome configurations has brought challenges to its assembly [18]. By October 2022, there were 525 plant mitogenomes in the NCBI organelle database (https://www.ncbi.nlm.nih.gov/genome/organelle/), fewer than nuclear genomes.

Grass (Poaceae) mitogenomes have been assembled from about 23 species including some cultivars from crops such as rice, barley and wheat, making Poaceae species an important group for comparative mitogenome research [19–22]. The mitogenome can confer cytoplasmic male sterility [23, 24] with accessible restorer genes to enable seed multiplication: in rice [25] and maize [26], mitogenomes of several male sterile lines have been reported. The mitogenome is thus used for breeding and generating F_1_ hybrid seeds.

There is extensive sequence migration between the cellular genomes in higher plants during their evolution and divergence. Specifically, the mitogenome includes abundant foreign sequences, including mitochondrial plastid DNAs (MTPTs) and mitochondrial nuclear DNAs (MTNUs) and retroelement fragments. We aim to generate an assembly of the *A. longiglumis* mitochondrial genome using a hybrid strategy with long, single-molecule sequencing to allow the resolution of alternative, large and complex structures [17, 27] and correction by deep sequencing and high-quality short reads. We then aim to characterize gene and repeat content, and the presence of sequence exchanges between plastomes and nuclear genomes, putting features into an evolutionary and phylogenetic context.

## Materials and methods

### Plant material and genome sequencing

Plants of *Avena longiglumis* Durieu (ALO) (accession PI 657,387; US Department of Agriculture, Beltsville, https://www.ars-grin.gov/, collected from the wild in Morocco in 1989 c. 500 m from the Mediterranean coast near Moulay Bousselham) was used for genome sequencing. After growing in South China Botanical Garden Greenhouse at 25 °C, 16 h light/8 h dark with 70% relative humidity for four weeks, plants were moved outside under natural daylight conditions (dry season in Guangzhou). Flowering specimens were identified by Qing Liu and vouchers were deposited in the South China Botanical Garden Herbarium (code IBSC).

Genomic DNA for Illumina mate-pair sequencing was extracted using the DNeasy Plant Mini Kit (Qiagen) from the 8-week-old leaves of ALO seedlings. A DNA library with an insert size of 350 bp was constructed following the manufacturer’s protocol [28], and was sequenced using the Illumina NovaSeq 6000 platform, giving 201.9 Gb (gigabases) of raw data from 673,011,605 raw reads. Clean data were obtained by using Trimmomatic [29] removing low-quality sequences, defined by a quality value of Q < 19 for more than 50% of the total bases or sequences in which more than 5% of bases were “N”.

For ONT PromethION library construction and long read sequencing, genomic DNA was extracted from 3-week-old leaves of ALO seedlings using the Qiagen 695 Genomic DNA Extraction Kit. The DNA libraries were quantified using a Qubit 3.0 Fluorometer (Cat. E33216, Invitrogen, USA) and loaded into 12 lanes of a PromethION, R9.4.1 flow cell (Oxford Nanopore Technologies, UK) for SMRT (single molecular real-time) sequencing. In total, 49.6 Gb of sequences in 2,131,575 reads were obtained.

### Plastid and mitochondrial genome assembly

For plastome assembly, the Illumina paired-end reads were assembled into contigs using the GetOrganelle v.1.7.3 pipeline [30] with the parameters of ‘-R 15 -k 21,45,65,85,105 -F embplant_pt’ recommended by the developer. We initially de novo assembled the mitogenome of *A. longiglumis* using the Nanopore raw long-reads with Flye v.2.9-b1774 [31] with the default settings. GFA format files from the assembly results of Flye were visualized using Bandage [32]. For all assembled units, we used makeblastdb to build the database of *A. longiglumis*. Subsequently, mitochondrial genes extracted from *Oryza sativa* indica Group (NC_007886.1) mitogenome were used as query sequences to identify the unitig graph containing mitochondrial genes and sequences were compared with the nucleotide database [33] (see below for annotation details).

Considering that there are regions of mitogenome that are homologous to chloroplast sequences, it is likely that they are replaced by their chloroplast counterparts during polishing. Therefore, we mapped the Illumina short-reads to the mitogenome assembly by Nanopore long-reads using BWA and SAMtools v.0.1.19 [34, 35], and all unmapped reads were excluded. BEDTools [36] were used to transform BAM files containing mapped reads into FASTQ format files. We obtained about 52 Mb of mitochondrial reads from 201.9 Gb raw data, which also contained chloroplast homologous sequences. We used the same processing strategy of the chloroplast sequences to screen the Nanopore long-reads to obtain mitochondrial reads and exclude nuclear and plastid reads with Minimap2 [37].

Subsequently, all processed long-reads and short-reads were used as input files for hybrid assembly by using Unicycler [38] with default settings. Unicycler will call Spades [39] to assemble Illumina short-reads, and repetitive DNA sequences encountered during assembly will be resolved using long-reads. Then, four circular contigs (isoforms) were generated, representing the complete mitochondrial genome of *A. longiglumis* with a graph showing alternative configurations. We found that the four contigs have long repeated sequences within each other. Finally, a master circular genome was generated by merging them manually.

### Genome annotation

CPGAVAS2 [40] was used for the annotation of *A. longiglumis* plastome with the third option of a custom reference, using the previously released GenBank format file of *A. longiglumis* plastome (NCBI accession number MK336391.1 [9]).

We first downloaded RNA-seq data from NCBI SRA database with the accession numbers PRJNA735431[12] and PRJNA838431 [14]. For the annotation of protein-coding genes (PCGs) and rRNA genes in the mitogenome, we used GeSeq [41] to annotate the assembled mitogenome of *A. longiglumis* with two reference mitogenomes from GenBank: *Oryza sativa* (NC_007886.1) and *Liriodendron tulipifera* (NC_021152.1). Subsequently, the web-based tool public MITOFY [42] was used to identify genes. Annotation was manually edited by Apollo [43]. All transfer RNA genes were confirmed by tRNAscan-SE with default settings [44] and compared with studied tRNA genes previously [45]. Based on sequence similarity, we identified tRNA genes that migrated from plastome and tRNA genes that were native to mitochondria. We did not include those tRNA genes with no homology in previous studies. Finally, we drew the genome map using OGDRAW [46].

### Homologous sequences between mitochondrial, plastid and nuclear genomes

To identify DNA fragments that may be transferred to the mitochondria from the plastid and nuclear genomes, we compared the three genomes using makeblastdb and BLASTn program [33] with the following parameters: ‘-evalue 1*e*-5, -word_size 9, -gapopen 5,—gapextend 2, -reward 2, -penalty -3, and -dust no’. The plastome assembled here (based on long-molecule sequences), and the nuclear genome of *A. longiglumis* downloaded from NCBI with the accession number of CM042677.1-CM042683.1 [12], OU342747.1-OU342753.1 [13] and SRR19279532-SRR19279533 [14] were used as reference in this study. For MTNUs, we only keep results with length longer than 1,000 bp.

### Analysis of repeat elements

Simple sequence repeats (SSRs) were identified using the web-application MISA [47] (https://webblast.ipk-gatersleben.de/misa/) for the assembled mitogenome, with parameters of the minimum numbers of mono-, di-, tri-, tetra-, penta-, and hexanucleotides set as 10, 5, 4, 3, 3, and 3, respectively. Additionally, forward, reverse, palindromic, and complementary repeat sequences were identified by REPuter (https://bibiserv.cebitec.uni-bielefeld.de/reputer/) with the following settings: hamming distance of three and minimal repeat size of 30 bp [48], and *e*-value is limited to less than 1*e*-05. Tandem Repeats Finder [49] (https://tandem.bu.edu/trf/trf.html) is used to detect the tandem repeats of the mitogenome.

### Identification of RNA editing sites in protein-coding region of mitochondrial genes

Two published RNA-seq datasets in the SRA database (https://www.ncbi.nlm.nih.gov/sra/; accession numbers SRR19216412 to SRR19216415 and SRR14760587 to SRR14760588) [12, 14] were combined to identify the RNA editing sites in mitochondrial PCGs. The RNA-seq data were mapped onto sequences of PCGs by using Bowtie2 [50] with the parameters: ‘-f -a -m 20 –al Reads_aligned –un Reads_unaligned’. We performed three runs with the mismatched numbers set to 3, 5, and 7, in order to ensure the excessive RNA editing events detected by enough mapping results; The anomalies results were checked by Tablet [51]. We used BCFtools [52] to call single nucleotide polymorphism sites (SNPs), which were considered as RNA editing sites. A standard that each RNA editing site was set to cover by at least 20 reads, and editing events had to occur in at least ten reads.

### Phylogenetic inference

The mitogenomes of 28 Poaceae accessions of 23 species downloaded from NCBI were used to construct a phylogenetic tree, with two other monocotyledonous species, *Cyperus esculentus* (Cyperaceae) and *Phoenix dactylifera* (Arecaceae) as outgroups (Additional file 2: Table S1). To ensure comparisons were valid, these mitogenomes were re-annotated using former described tools. A total of 30 orthologous mitochondrial genes among the analyzed species were identified and extracted by PhyloSuite v.1.2.1 [53]. The corresponding nucleotide sequences were aligned by MAFFT v.7.450 [54]. Next, these aligned sequences were concatenated and used to construct the phylogenetic trees. The consensus sequences included 31,510 nucleotide sites. The maximum likelihood (ML) method implemented in RAxML v.8.2.4 [55]. The parameters were “raxmlHPC-PTHREADS-SSE3 -f a -N 1000 -m GTRGAMMA—× 551,314,260 -p 551,314,260”. The bootstrap analysis was performed with 1,000 replicates. Bayesian inferences (BI) analysis was performed by MrBayes v.3.2.6 [56] with the Markov Chain Monte Carlo method for 200,000 generations and sampling trees every 100 generations. The first 20% of trees discarded as burn-in, the remaining trees were used to generate a consensus tree.

## Results

### Mitochondrial genome structure of *Avena longiglumis*

Based on the hybrid assembly of Unicycler using Illumina paired short reads and ONT PromethION long reads, a graphical representation of the *A. longiglumis* mitogenome is obtained. It contains 36 unitigs (high-confidence sub-contigs, Additional file 1: Figure S1), which show duplications and the alternative tiling paths. Length and sequencing depth of each unitig are given in Additional file 2: Table S2).

There are long repetitive sequences between the contigs which may potentially generate the multiple isoforms via genome recombination (Additional file 1: Figure S1, Additional file 2: Table S2). The alternative configurations (isoforms) are supported by Oxford Nanopore long reads. In order to describe the mitogenome succinctly, we simplify the four contigs into a master circular genome based on the shared repeats. The detailed solution and schematic diagram can be found in Additional file 1: Figure S2.

The total length of the mitogenome of *A. longiglumis* is 548,445 bp during the reconstruction of a single circle mediated by repeats. The GC content is 44.05%. Accuracy of the mitochondrial genome bases was confirmed by mapping the Illumina paired short read raw data (average 56-fold coverage) onto the assembled reference mitogenome (Additional file 1: Figure S3).

### Gene content and repeat elements of mitochondria

We identify a total of 65 mitochondrial native genes (Table 1, Additional file 2: Table S3), including 39 mitochondrial native protein-coding genes (35 are unique), eight mitochondrial native rRNA genes (three are unique), and 18 mitochondrial native tRNA genes (11 are unique). Furthermore, there are ten complete plastid-derived genes, including one protein-coding gene (*rpl2*) and nine tRNA genes (eight are unique and there are two copies of *trnC*-GCA). The multiple dispersed and tandemly repetitive DNA elements are characterised (Additional file 2: Tables S4, S5 and S6).

**Table 1:** Gene composition of the mitogenome of *A. longiglumis*. For lengths and positions of genes see Additional file 2: Table S3

	Group of genes	Genes (Number of copies in parentheses if > 1)
Protein coding genes (PCGs)	ATP synthase	*atp1* (× 3), *atp4* (× 2), *atp6*, *atp8*, *atp9*
	NADH dehydrogenase	*nad1*, *nad2*, *nad3*, *nad4*, *nad4L*, *nad5*, *nad6*, *nad7*, *nad9*
	Cytochrome c biogenesis	*cob*
	Ubiquinol cytochrome c reductase	*ccmB*, *ccmC*, *ccmFC*, *ccmFN*
	Cytochrome c oxidase	*cox1*, *cox2*, *cox3*
	Maturases	*matR*
	Transport membrane protein	*mttB*
	Large subunit of ribosome	*rpl5*, *rpl16*, *rpl2* (cp)
	Small subunit of ribosome	*rps1*, *rps2*, *rps3*, *rps4* (× 2), *rps7*, *rps12*, *rps13*, *rps14*
Ribosomal RNAs		*rrn5* (× 3), *rrn18* (× 3), *rrn26* (× 2)
Transfer RNAs		*trnC-GCA* (× 2, cp), *trnD-GUC* (× 2), *trnE-UUC* (× 3), *trnF-GAA* (cp), *trnfM-CAU* (× 4), *trnH-GUG* (cp), *trnI-CAU*, *trnK-UUU*, *trnM-CAU* (cp), *trnN-GUU* (cp), *trnP-UGG* (× 2), *trnP-UGG* (cp), *trnQ-UUG*, *trnS-GCU*, *trnS-GGA* (cp), *trnS-UGA*, *trnV-GAC*, *trnW-CCA* (cp), *trnY-GUA*

The numbers in parentheses represent copy numbers of genes, and ‘cp’ represent the plastid-derived genes

**Fig. 1:**
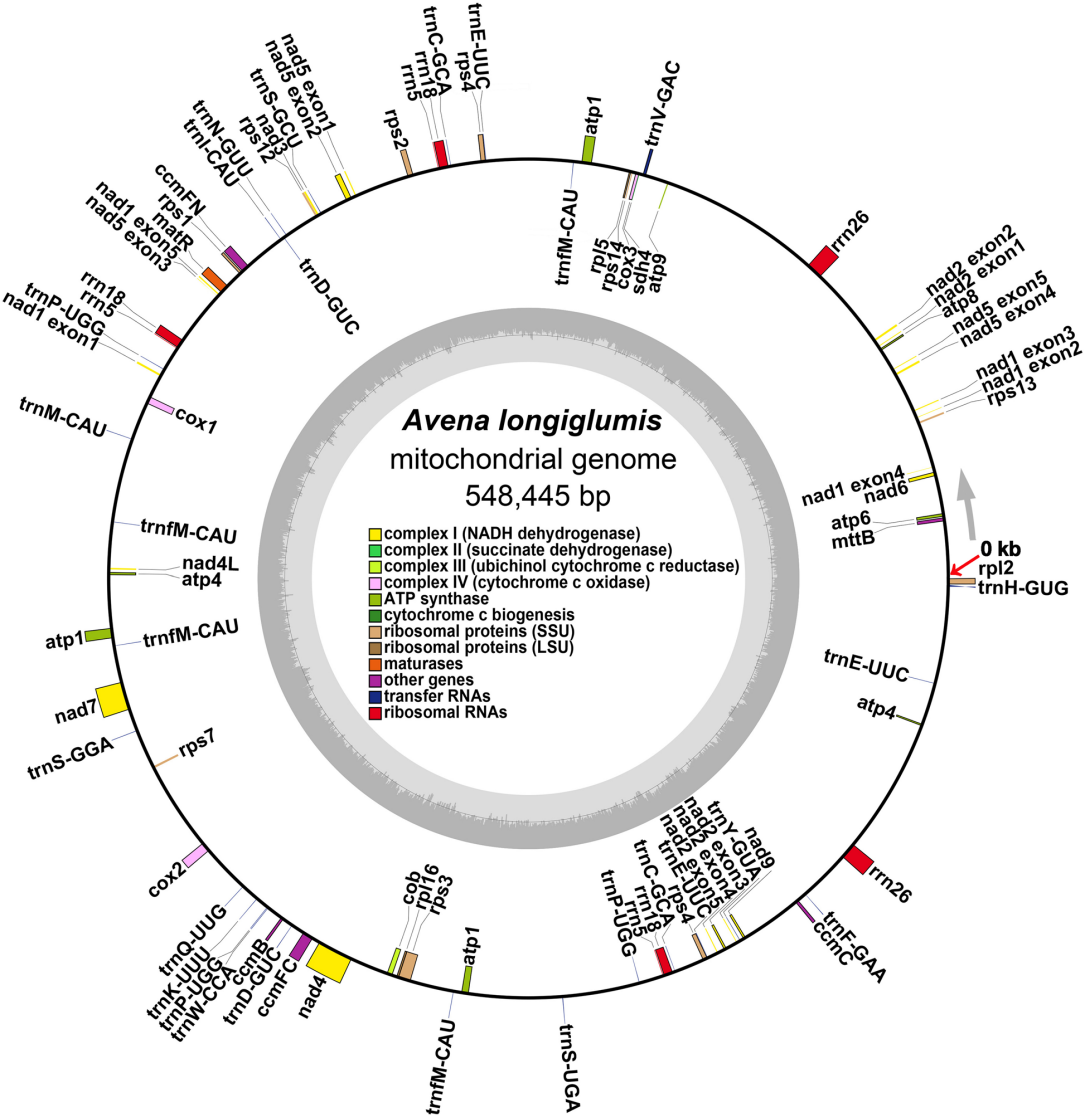
The circular mitogenome maps of *A. longiglumis.* The gene contents (see Table 1) of mitogenome with the starting point marked by red arrow and the direction of positive strand marked by the gray arrow. Genes transcribed clockwise and counter-clockwise are drawn on the inside and outside of the circles (location details see Additional file 2: Table S3). Genes belonging to different functional groups are color-coded

The protein-coding genes (PCGs) (Table 1, Fig. 1) include 24 unique core mitochondrial genes: five subunits of ATP synthase, nine subunits of NADH dehydrogenase, four cytochrome C biogenesis genes, three cytochrome C oxidase genes, and one transport membrane protein, maturase, and ubiquinol cytochrome C reductase, respectively. Ten variable mitochondrial genes are identified, including two large subunits of ribosome proteins and eight small subunits of ribosome proteins. Among these, *atp1* has three copies, *atp4* and *rps4* have two copies of each one, and there are three rRNA genes (with 3 copies of *rrn5* and two copies *rrn18* and *rrn26* of each one). A total of 18 unique (total 27) tRNA genes are identified; six tRNA genes have multiple copies, e.g. *trnC-GCA* and *trnD-GUC* have two copies for each one (Table 1, Additional file 2: Table S3).

**Fig. 2:**
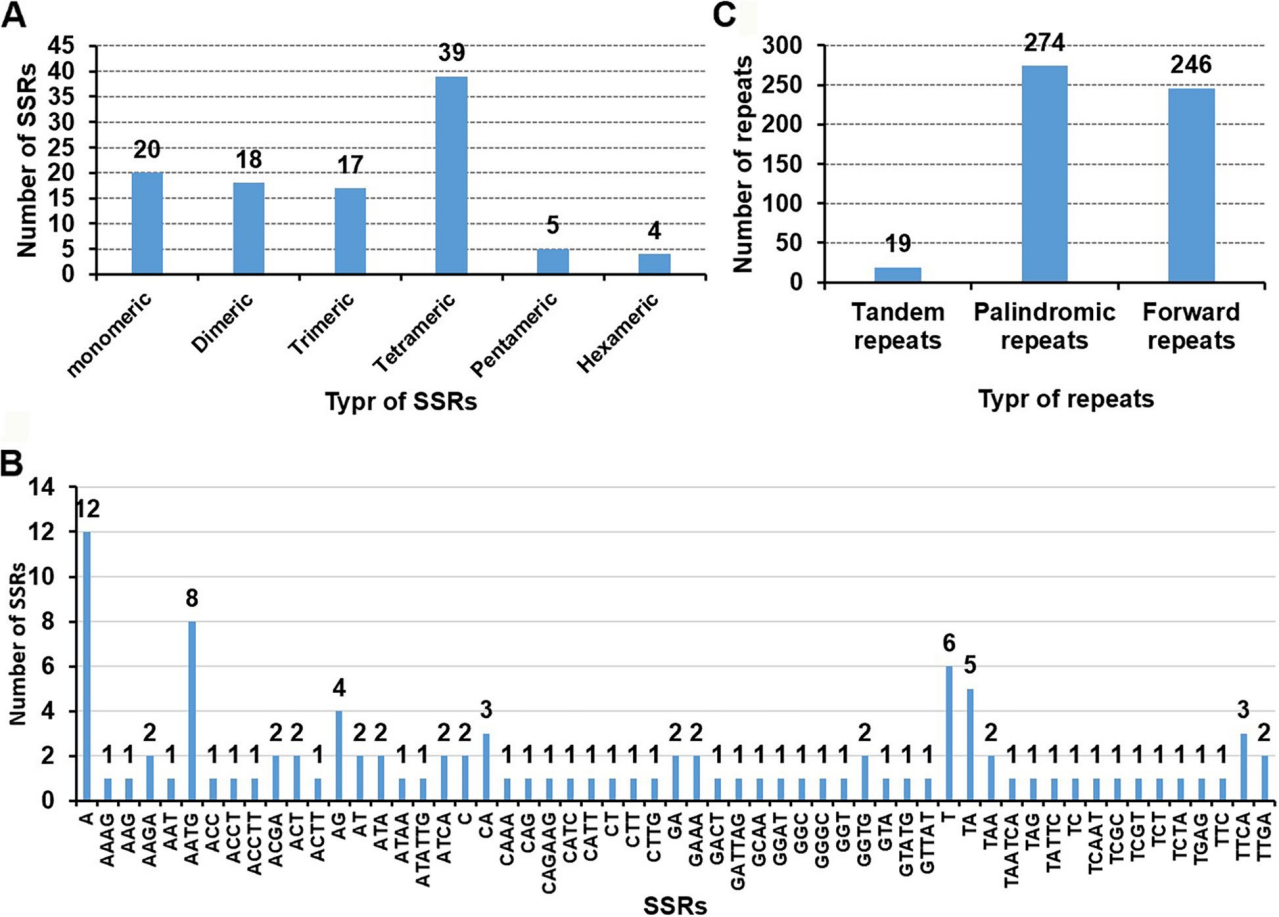
The simple sequence repeats (SSRs), tandem repeats, and dispersed repeats identified in the mitogenomes of *A. longiglumis*. **A** The identified SSRs on the reference mitogenomes of *A. longiglumis*. Each column represents different nucleotide repeat units. The numbers of repeats in each category are shown on the top of corresponding columns (see Additional file 2: Table S4). **B** The type and number of SSRs. **C** Tandem repeats (with repeat unit ≥ 12 bp, see Additional file 2: Table S5) and dispersed repeats (≥ 30 bp, see Additional file 2: TableTable S6) identified on four contigs

We find 103 simple sequence repeats (SSRs) in the mitogenome (Fig. 2A, Additional file 2: Table S4). Tetrameric repeats accounted for 38% of the SSRs (39 out of 103) compared to 20 or fewer for monomeric, dimeric and trimeric repeats; or 5 or fewer for hexamers and pentamers. For these SSRs, there are 18 stretches of the mononucleotide A/T, respectively. In addition, there are 5 dimers of TA and 8 tetramers of AATG (Fig. 2B).

**Fig. 3:**
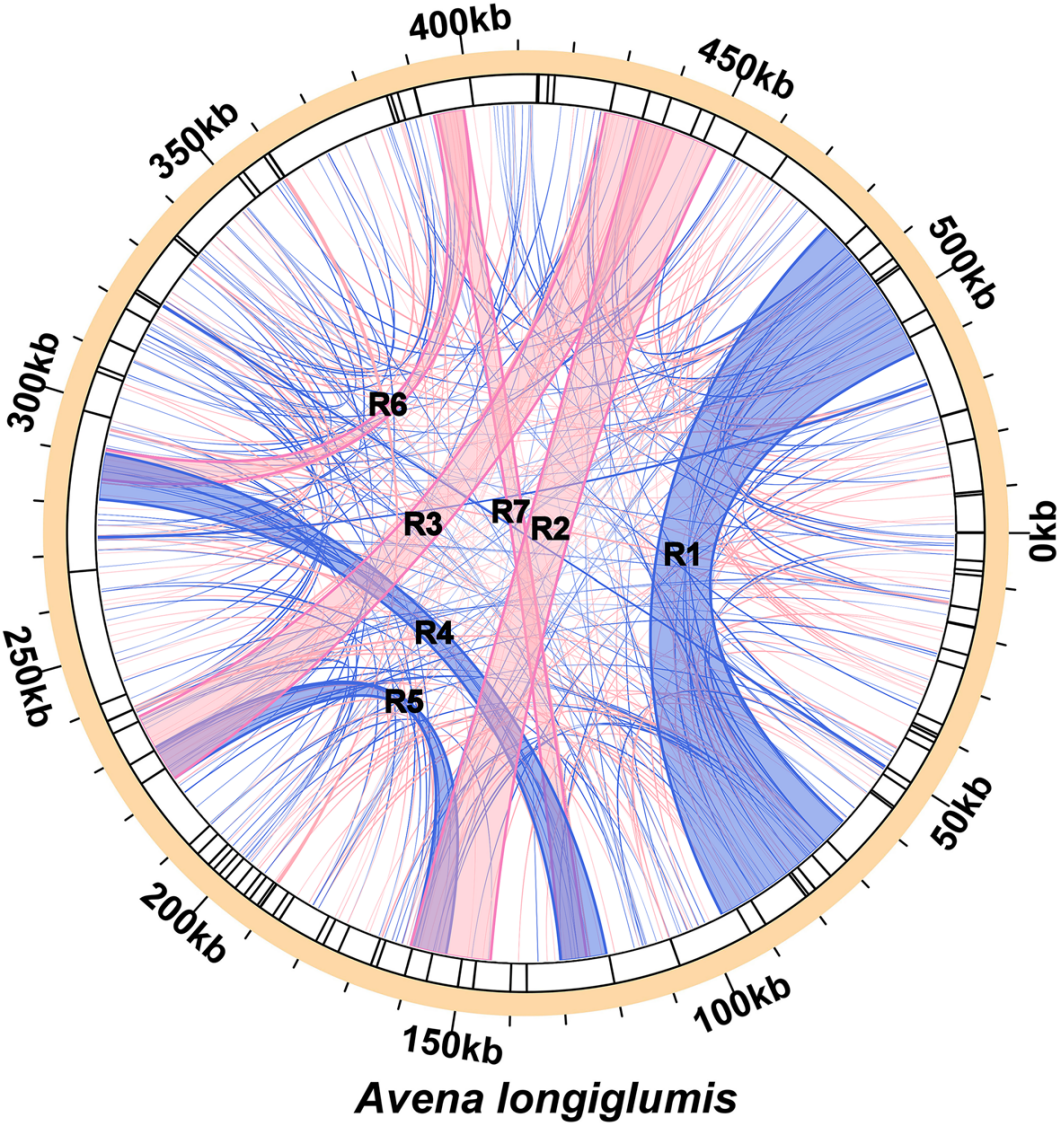
The distribution of dispersed repeats among the mitogenome of *A. longiglumis*. Arcs connect similar repeats within the mitogenome; blue arcs represent 246 forward repeats and the red represents 274 palindromic repeats (see Additional file 2: Table S6). Seven long repeats over 5,000 bp in length are labelled

We detect 19 long tandem repeats (> 11 bp repeat unit) (Fig. 2C, Additional file 2: Table S5). Duplicated non-coding sequences (≤ 30 bp) are more frequent than SSRs and tandem repeats: a total of 520 duplicated sequences are detected, among which 52.69% (274) are palindromic repeats, and 47.31% (246) are forward repeats (Additional file 2: Table S6), accounting for 42.5% (233,082 bp, elimination of overlapping region) of the 548 kb mitogenome of *A*. *longiglumis*. Most of these duplicated sequences (93%) are less than 100 bp in length, but three are much longer (R1 is 31,235 bp, R2 is 16,549 bp, and R3 is 14,514 bp), and five (R4-R7) between 5 and 10 kb long (Fig. 3, Additional file 2: Table S6).

### Sequence exchange between genomes: Identification of MTPTs and MTNUs

**Fig. 4:**
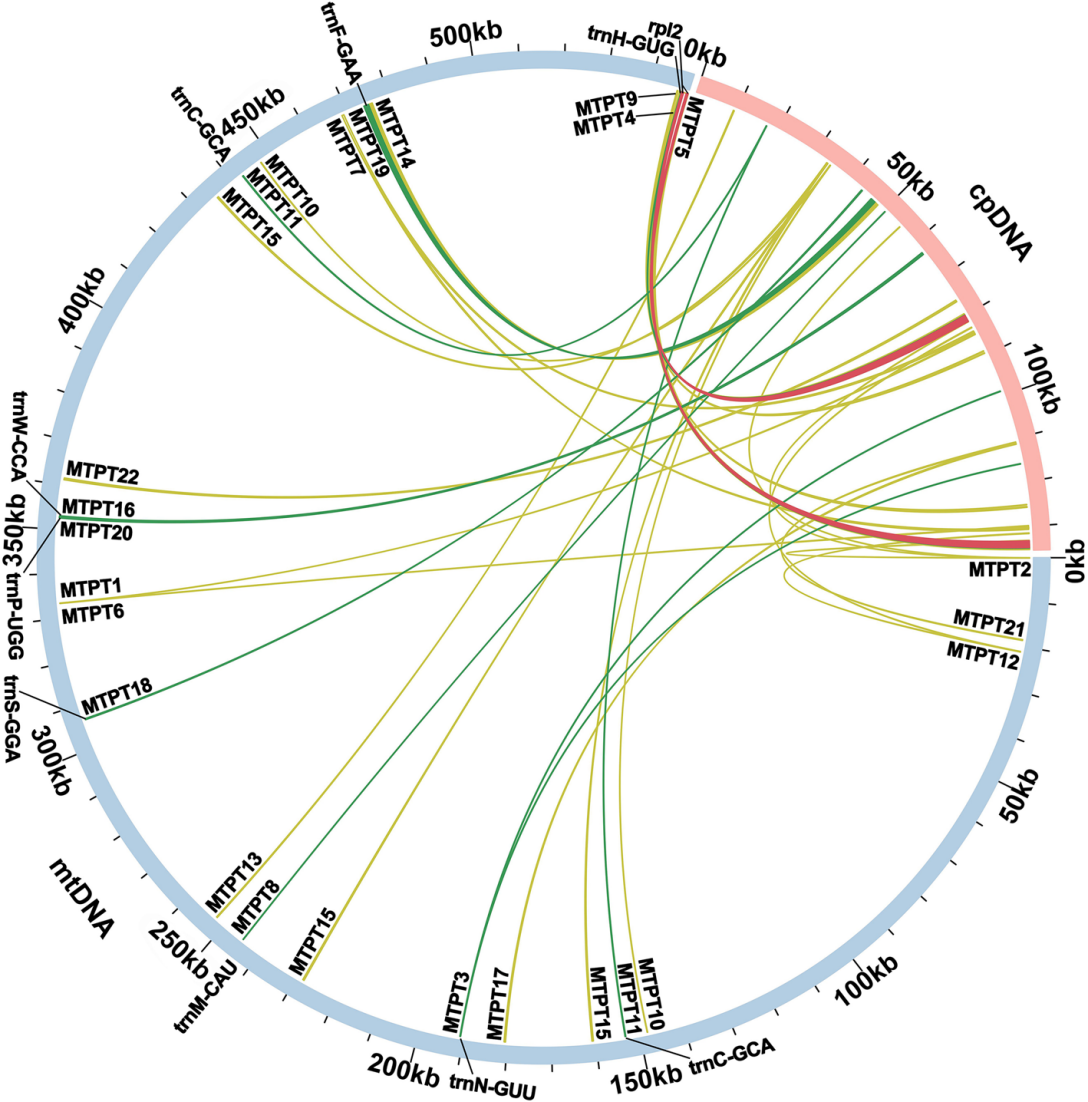
Schematic representation of the distribution of homologous sequences between mitogenome and plastome of *A. longiglumis*. Mitochondrial plastid DNAs (MTPTs) on the mitogenome are few and short in length. The outer circle of blue and pink section represents the mitogenome (mtDNA) and the plastome (cpDNA), respectively. The scale is 10 kb, marked on the genome. The yellow-green arcs in the middle represent the 22 MTPTs identified (please see Additional file 2: Table S7). We highlight the arcs in dark green and red if MTPT contained complete tRNA genes and protein-coding gene, respectively. They represent genes that migrated from the plastome to the mitogenome, including nine tRNA genes (eight are unique and there are two copies of *trnC-GCA*) and one protein-coding gene (*rpl2*). The homologous sequences located in the IR region of plastome was counted only once

We identify homologous sequences between the *A. longiglumis* mitogenome and the plastid and nuclear genomes. For MTPTs, the results show 22 homologous fragments between mitochondrial and plastid genomes (Fig. 4), the longest being 1,971 bp (MTPT5), followed by 1,125 bp (MTPT19). The total length of these fragments is 8,207 bp. These fragments contained some genes, including nine complete plastid-origin tRNA genes (*trnF-GAA*, *trnP-UGG*, *trnS-GGA*, *trnC-GCA*, *trnW-CCA*, *trnH-GUG*, *trnM-CAU*, *trnN-GUU* and *rpl2*), and nine plastid gene fragments, like *ndhA*, *ndhK*, *ndhJ*, *rpl14*, *atpA*, *rps19*, *rpl23*, *rps7* and *psbD* (Additional file 2: Table S7). Furthermore, a total of 16 sequence fragments of conserved protein domains (reverse transcriptase, RNaseH and integrase) of *copia* and *gypsy* nuclear retroelements are detected in the *A. longiglumis* mitogenome.

We detect a total of 502,359 bp (91.60%), 468,212 bp (85.37%) and 456,990 bp (85.32%) MTNUs in three nuclear genome assemblies of *A. longiglumis* [12–14], respectively (Additional file 2: Table S8). Most of the mitochondrial fragments are 1–2 kb long and only few more than 50 kb; they are dispersed throughout all seven chromosome assemblies of the nuclear genome.

### RNA editing and gene structures

**Fig. 5:**
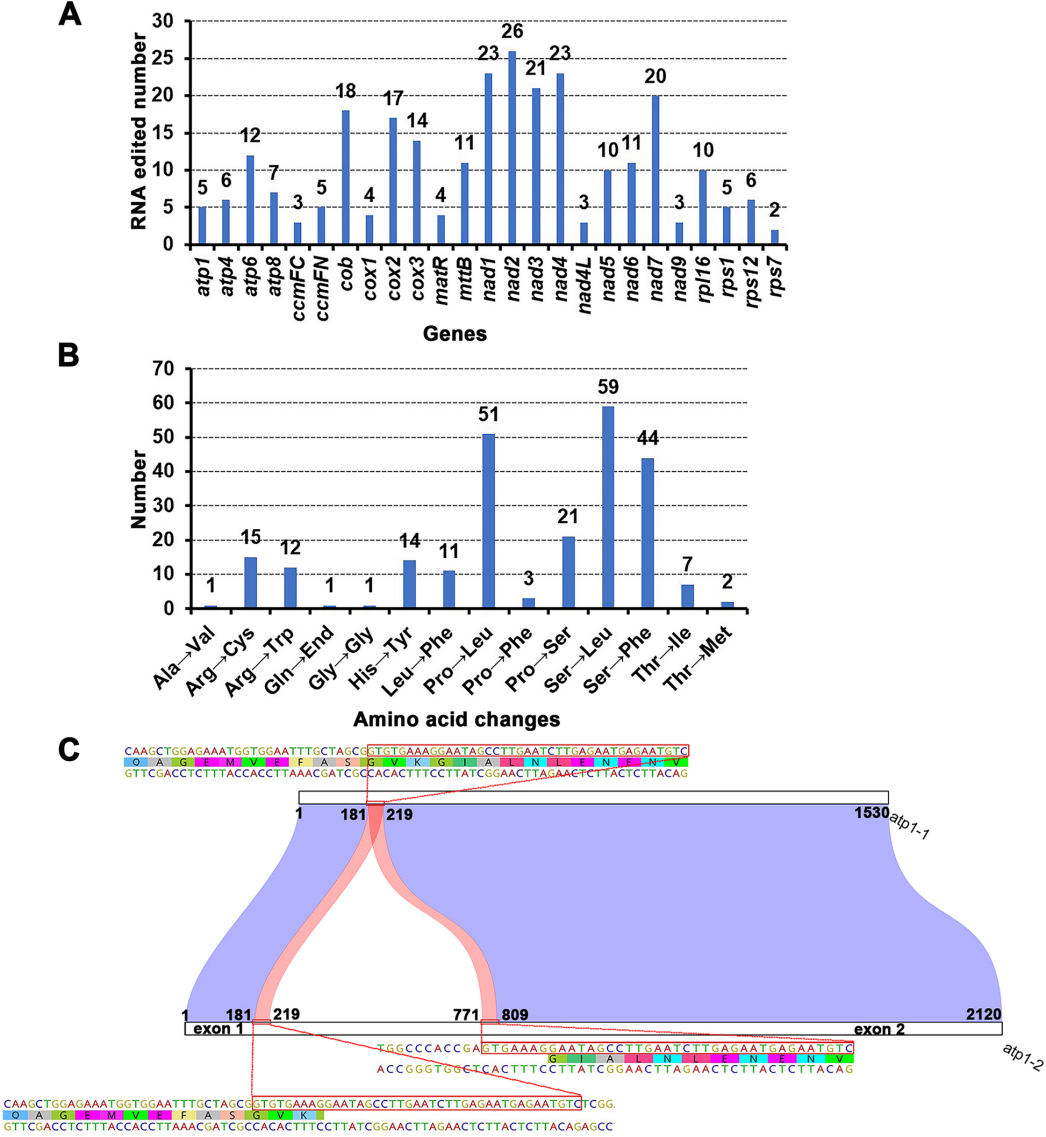
Characteristics of the RNA editing sites identified in protein-coding genes (PCGs) and schematic structure of *atp1* gene of the *A. longiglumis* mitogenome. **A** Frequency of RNA editing sites identified in each PCGs. **B** Frequency of amino acid changes caused by RNA editing. **C** Schematic diagram of the *atp1* gene structures

We identify the RNA editing sites of mitogenome-encoded protein coding genes. The results show that a total of 25 mRNAs of PCGs is identified RNA editing events, and all these edited sites are C to U editing. For these 25 genes, we identify 269 high-quality RNA editing sites (Fig. 5A, Additional file 2: Table S9; *nad2* has 26 editing sites, *nad1* and *nad4* have 23 of each one, *nad3* has 21 and *nad7* had 20).

The 269 editing sites involves 258 codons and resulted in 242 amino acid changes (Fig. 5B). Three edits alone accounted for more than 63% of the amino acid changes: serine replacement with leucine occurred 59 times, while substitution of proline with leucine and serine with phenylalanine are identified 51 and 44 times respectively (Fig. 5B). Though supported by relatively low coverage sequencing reads, we find RNA editing events for the *ccmFC* gene to create stop codon (Additional file 1: Figure S4A). By comparing it with the other two species, *Arabidopsis thaliana* and *Nicotiana tabacum*, we suggest that this RNA editing might be meaningful to maintain the conserved length of the protein (Additional file 1: Figure S4B).

Eight of the 34 protein coding genes harbour introns: four genes contain one intron (two copies of *atp1* gene, *ccmFC* and *cox2*), one gene contains three introns (*nad4*), and four genes contain four introns (*nad1*, *nad2*, *nad5* and *nad7*). The *atp1* gene has two different gene structures; one copy has no intron, while the other two copies contain one intron (Fig. 5C). However, the genuine identity of this intron needs to be treated cautiously since since introns have not been reported for the *atp1* gene in angiosperms. The insertion of this unknown intron sequence might be associated with a 39 bp small repeat sequence, perhaps introduced during genome repair.

### Phylogenomic analysis

**Fig. 6:**
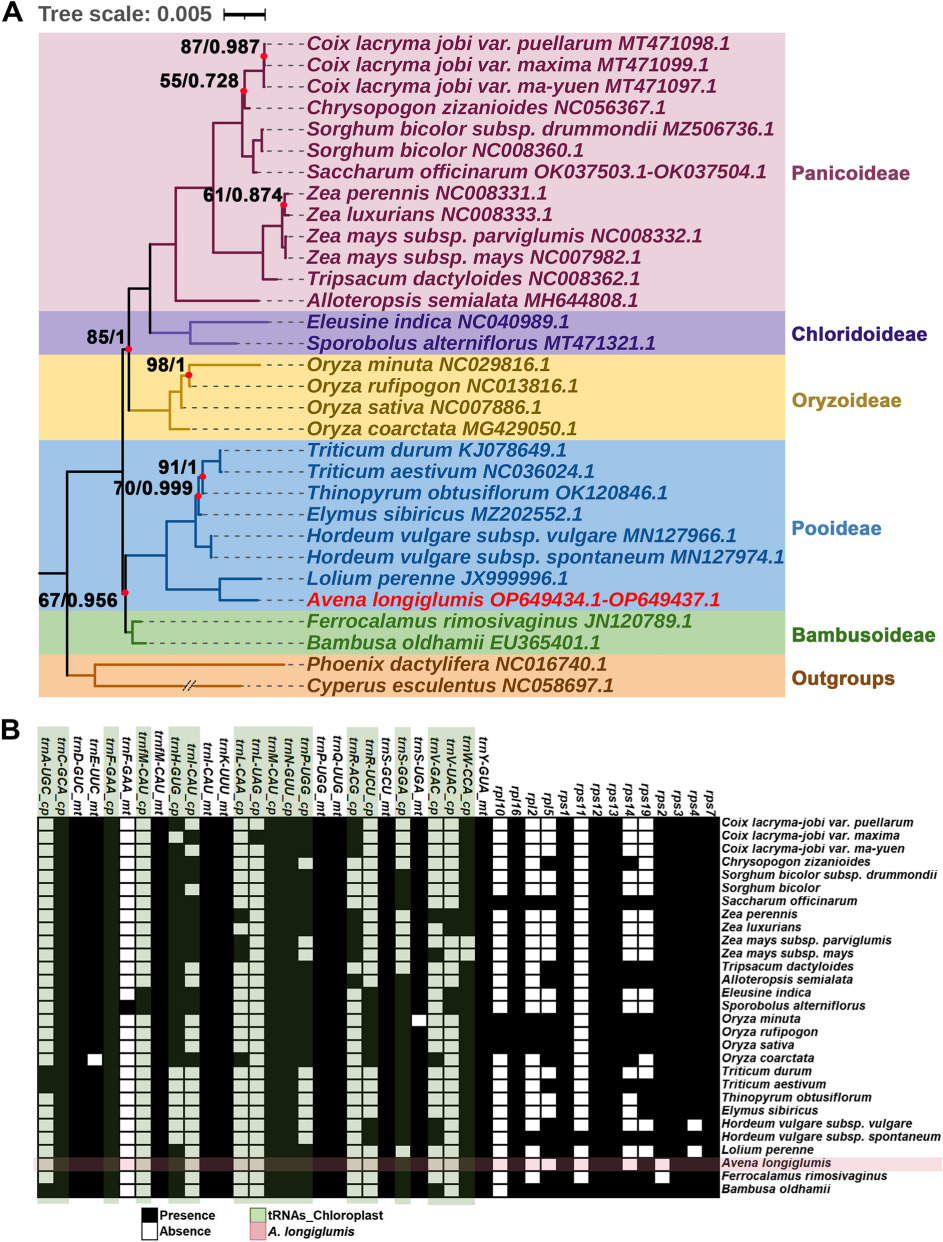
The phylogenetic relationships and gene distribution in mitogenomes of *A. longiglumis* compared with 23 Poaceae species. **A** Maximum Likelihood (ML) and Bayesian Inference (BI) tree reconstructing the phylogenetic relationships of mitochondrial genomes. The subfamilies of Poaceae (colored blocks) are only weakly resolved in red nodes. The tree is based on nucleotide sequences of 30 conserved mitochondrial PCGs (*atp1*, *atp4*, *atp6*, *atp8*, *atp9*, *nad1*, *nad2*, *nad3*, *nad4*, *nad4L*, *nad5*, *nad6*, *nad7*, *nad9*, *ccmB*, *ccmC*, *ccmFC*, *ccmFN cox1*, *cox2*, *cox3*, *cob*, *mttB*, *matR*, *rpl16*, *rps1*, *rps3*, *rps7*, *rps12* and *rps13*). The ML topology is indicated with ML bootstrap support values and BI posterior probabilities at each node; red nodes represent bootstrap support less than 100% (ML) or 1 (BI). Two other monocotyledonous species (*Cyperus esculentus* and *Phoenix dactylifera*) are used as outgroups. **B** Presence of non-core mitochondrial genes in Poaceae where filled squares represent presence of at least one complete copy. While some features are shared in subfamilies, there is substantial variation; *A. longiglumis* (red shading) shows characteristic differences. _cp (green): tRNA genes that migrated from plastome; _mt represents mitochondria-native tRNA genes

Phylogenetic analyses based on the mitochondrial genomes yield a phylogeny of Poaceae species with eight nodes having Maximum Likelihood (ML) bootstrap support values < 99% or Bayesian Inference (BI) posterior probabilities < 1 (Fig. 6A). Given the paucity of mitogenome data, the analysis presented here is based only on the available Poaceae mitogenomes (for just 24 species, Additional file 2: Table S1) of the more than 11,000 species in Poaceae family [57].

We compare gene content of mitogenomes of *A. longiglumis* with other 28 mitogenome accessions of 23 Poaceae species. Three unique rRNA genes and 24 core mitochondrial genes [58] are present in all the analyzed Poaceae species. For the tRNA genes, we examine the origin based on sequence similarity (Fig. 6B; see detail in Additional file 2: Table S10) between genomes and species [45]. Five of 17 tRNA genes originating from plastids (*trn_cp*) presented in most mitogenomes of Poaceae: *trnC-GCA_cp*, *trnF-GAA_cp*, *trnM-CAU_cp*, *trnN-GUU_cp* and *trnW-CCA_cp* are identified in at least 27 out of 29 mitochondrial genomes. In contrast, plastid-derived *trnA-UGC_cp*, *trnfM-CAU_cp*, *trnL-CAA_cp*, *trnL-UAG_cp* and *trnV-UAC_cp* are found in four or fewer taxa. A total of eleven mitochondrial native tRNA genes are identified. Ten are present in most species, but *trnF-GAA_mt* was detected in only *Sporobolus alterniflorus* (syn. *Spartina alterniflora*). Four mitochondrial native tRNA genes identified in other angiosperms are lost in Poaceae, *trnC-GCA*, *trnG-GCC*, *trnV-UAC* and *trnW-CCA*.

For the variable PCGs (large and small ribosomal proteins), eight are shared among 22 or more of the 24 species. Three frequent mitochondrial genes are unidentified in the analyzed species, including *rps10*, *sdh3* and *sdh4*. Genes have lost to varying degrees in the various species and many gene losses evidently occurred several times in the grass phylogeny.

## Discussion

We present a complete sequence assembly, 548,445 bases long, of the *Avena longiglumis* mitochondrial genome (Fig. 1) and analyze repeats and the gene content in a phylogenetic context. However, the multi-isoform (multi-chromosomal) architecture is highly possible, because Unicycler obtained four independent and circular contigs based on resolving repeats using long-reads (Additional file 1: Figure S1). The results are consistent with our understanding of the mitogenomes of higher plants (especially angiosperms), showing possible rearrangements and genome recombination [59, 60]. The size is within the range reported in plant species, although longer than the average for land plants of 394,900 bases [61]. Kozik et al. [17] suggest that the plant mitogenomes should be presented as multiple sequence units showing their variable and dynamic connections rather than as circles. In our case, the complex assembly configurations, which are the result of recombination between repeats, are shown in Additional file 1: Figure S1A and S1B. For *A. longiglumis* mitogenome, we suggest that seven pairs of long dispersed repeats (R1 to R7, Fig. 3) are the key for the mitogenome recombination. Dispersed repeats have been reported to mediate the recombination of the mitochondrial genome resulting in various alternative configurations (isomers) [62, 63], with data from various plants including *Nymphaea colorata* [64], *Silene latifolia* [41], *Mimulus guttatus* [65], and *Ginkgo biloba* [66]. In the *Arabidopsis thaliana* mitogenome, two large repeats (6.0 and 4.2 kb in size in the 368 kb genome) are constantly recombining and lead to multiple alternative structures [67].

In Poaceae species, there is only limited research with relatively  few reports of mitochondrial genomes and their complexity [68]. Some assemblies have reported one, or several, circular contigs describing the whole mitogenome, enabling the data to be used to address key questions about gene content and gene polymorphisms, but not repeats nor genome structure. Sugarcane, for example, has two circular contigs as the complete mitogenome [68]. As we find in *A. longiglumis*, rearrangements involving inversion, translocation, fusion, and fission have also been shown in mitochondrial genomes of rice, with various frequencies of alternatives [69]. For most cultivated grasses, the complex and polyploid large nuclear genome, and low copy number of mitochondria, pose a challenge to the amount of data being able to be sequenced [19, 21]. Further long-molecule sequence coverage and PCR amplification across recombination breakpoints marking alternative configurations, possibly with enrichment of mitochondria based on differential centrifugation and subsequent DNA extraction for sequencing (as often used in human cell and tissue [70]), may be helpful.

In detailed analysis of sequence data, we find evidence for multiple RNA edits (Fig. 5). Notably, creation of stop codons via RNA editing events was in a few transcripts of *ccmFC* gene, with two of three reads supporting an edit to add a stop codon in the gene (Additional file 1: Figure S3). This result is consistent with previous research with *ccmFC* [71], and editing maintaining the stability of the length of the protein product encoded by the *ccmFC* gene.

Exchange of sequence between mitochondrial genomes and those of the plastid—MTPTs— and nucleus—MTNUs—are ubiquitous in seed plants [72]. Studies of MTPTs in *A. longiglumis* here found relatively few plastid homologous sequences, representing only 8,207 bp or 1.5% of the mitogenome. This result shows sequence migration between these two organelle genomes. Large segments of MTPTs have been reported in many mitochondrial genomes. A total of 26.87 kb MTPTs were found in *Suaeda glauca* [73], accounting for 5.18% of its mitogenome. The large MTPTs are also found in *Salix suchowensis* [74]. The MTPTs were thought to broadly impact eukaryotic evolution and promote genetic diversity [75]. Although the MTPTs were shorter in length in *A. longiglumis*, some plastid genes are still transferred intact from the plastome to the mitogenome. There are eight tRNA genes. It is difficult to determine whether these plastid-derived tRNA genes have biological functions. Previous studies have shown that *trnH-GUG* (cp) and *trnM-CAU* (cp) might still be functional in plant mitogenomes, and they migrated into the mitogenome early in the evolution of the species [41, 76]. In contrast, neither mitochondrial native *trnM-CAU* nor *trnH-GUG* has been reported in higher plants. Another three migrated tRNA genes, *trnN-GUU* (cp), *trnP-UUG* (cp), and *trnW-CCA* (cp), possibly also are functional, as reported by Richardson et al. [77]. The *trnF-GAA* (cp) and *trnC-GCA* (cp), although sporadically migrated to the mitogenome in other angiosperms, were detected in almost all Poaceae species. These results suggest that the migration of these two plastid-origin tRNA genes into mitogenome is universal in Poaceae, with only a few exceptions. Moreover, *trnS-GGA* (cp) was found only in *A. longiglumis* and some Poaceae plants. As for some plastid-derived tRNA genes found in other Poaceae species, such as *trnfM-CAU* (cp) and *trnA-UGC* (cp) [78, 79]. Their migration is sporadic rather than universal, just like *trnS-GGA* (cp). They possibly have migrated along with the large plastid fragments recently, and this event is not universally shared among the family Poaceae. As for the variable PCGs, we found that only six of them are conserved in Poaceae. Besides this, two succinate dehydrogenases (*sdh3* and *sdh4*) are lost in all Poaceae species, which have been reported to transfer to the nuclear genome [80]. This result is a general characteristic of these taxa.

We identify large fragments of mitochondrial sequences in two sets of nuclear genomes, and find 85 to 92% of the mitochondrial sequence in the nuclear genome assemblies of *A. longiglumis* (Additional file 2: Table S8). The results are compatible with those from high-quality assemblies of *Arabidopsis thaliana* [67], although in *Arabidopsis*, the careful reassembly of the nuclear genome shows there is a single major insertion of a structurally complex MTNU representing more than one copy of most of the mitochondrial genome. Here, for *A. longiglumis*, we find many MTNUs of smaller size distributed over all nuclear chromosomes.

We detect multiple retroelement domains within the mitochondrial sequence of *A. longiglumis* (although no long stretches representing complete elements or even open reading frames, ORFs) showing the transfer of fragments from the nucleus. We speculate that the transferred sequence fragments may originate from episomal DNA copies of retroelements that have been reverse transcribed from RNA transcripts present in the cell and integrated via recombination of party homologous sequences (see Richert-Pöeggeler et al. [81]) contrasting with the MTNUs originating directly from the nuclear genome. In many species including *Arabidopsis*, numerous fragments of retrotransposons have been found integrated into the mitochondrial genome [82].

Within the family Poaceae, phylogenetic inference based on mitochondrial sequence places *A. longiglumis* as a sister to *Lolium perenne* and the tribe Triticeae (Fig. 6A). However, subfamily Pooideae (including *A. longiglumis*) is placed only as weakly resolved sister to Bambusoideae (67% bootstrap support), and the subfamily Oryzoideae is a weakly resolved (85% bootstrap support) sister to Chloridoideae, and Panicoideae. Nuclear [83] and plastid [84] phylogenies, and indeed morphological studies, universally show strong support for two major grass clades, BOP (Bambusoideae, Oryzoideae, Pooideae) and PACMAD (Panicoideae, Arundinoideae, Chloridoideae, Micrairoideae, Aristidoideae, and Danthonioideae; including the C_4_ photosynthesis grasses) in the family Poaceae. Neither the mitochondrial sequence gene phylogeny (Fig. 6A), nor shared losses or gains of non-core genes (Fig. 6B), support separation of the clades, despite their being universally accepted as a natural division. Thus, our tree shows why, in contrast to animals, plant mitochondrial sequences are not usable in routine phylogenetic investigations. More mitochondrial assemblies are required for detailed analysis of the evolutionary and phylogenetic implications (including consideration of reasons for the low bootstrap or MI support for deep branches) for this organelle, including also basal grass genera such as *Anomochloa*.

## Conclusions

Our reference mitogenome of *A. longiglumis* shows its complex structure and repeat-rich organization based on long-read sequencing, with important features of recombination and RNA editing for the important cereal crop relatives. These results provide an important model for grass evolution and an essential reference for mitochondrial-associated characters, not least male sterility, in cereal crop breeding.

## Supplementary Information

**Additional file 1: Figure S1.** Graphical assembly display of the mitogenome based on Unicycler using Illumina short reads and ONT long reads. **Figure S2.** The master circular mitogenome generated by manually merging the four contigs based on long repetitive sequences. **Figure S3.** Sequencing coverage based on Illumina short-reads. **Figure S4.** RNA-editing to create a stop codon of gene ccmFC.

**Additional file 2:** **Table S1.** Species and GenBank accessions used for phylogenetic analysis. **Table S2.** Characteristics of unitigs assembled by Unicycler. **Table S3.** Annotated genes and their locations. **Table S4.** SSRs identified in the mitochondrial genome of A. longiglumis. **Table S5.** Tandem repeat sequences identified in the mitochondrial genome of A. longiglumis. **Table S6.** Duplicated repeats (≥ 30 bp) identified in the mitochondrial genome of A. longiglumis. **Table S7.** The homologous DNA fragment identified among the mitochondrial genome and plastome of A. longiglumis. **Table S8.** The blastn results among the mitogenome (Query) and nuclear genome (Subject) of A. longiglumis. **Table S9.** RNA editing events identified in the mitochondrial protein coding gense of A. longiglumis. **Table S10.** tRNA genes identified in the mitochondrial genome of 29 Poaceae accessions.

## Data Availability

The raw sequencing data for the Illumina short-reads and Nanopore long-reads and the mitogenome sequences have been deposited in NCBI (https://www.ncbi.nlm.nih.gov/) with accession number: PRJNA838431, SAMN28422612, SRR19279518, SRR19279531. The accession number of master circular mitogenome is OQ450323 (https://www.ncbi.nlm.nih.gov/nuccore/OQ450323).

## Abbreviations

BI: Bayesian Inference
BLAST: Basic local alignment search tool
cp: Plastome
GC: Guanine-cytosine
ML: Maximum Likelihood
mitogenome: Mitochondrial genome
mt: Mitochondria contig
MTPTs: Mitochondrial plastid DNAs
MTNUs: Mitochondrial nuclear DNAs
NCBI: National Center for Biotechnology Information
PCGs: Protein-coding genes
SNPs: Single nucleotide polymorphism sites
SSRs: Simple sequence repeats
tRNAs: Transfer RNAs
