# Supplementary material for: The mitochondrial genome of the diploid oat Avena longiglumis
Source: BMC Plant Biol. 2023 Apr 26;23:218. doi: 10.1186/s12870-023-04217-8 (PMC10131481; doi:10.1186/s12870-023-04217-8)
Supplement: Supplementary file 1 — Additional file 1: Figure S1. Graphical assembly display of the mitogenome based on Unicycler using Illumina short reads and ONT long reads. Figure S2. The master circular mitogenome generated by manually merging the four contigs based on long repetitive sequences. Figure S3. Sequencing coverage based on Illumina short-reads. Figure S4. RNA-editing to create a stop codon of gene ccmFC. [file 12870_2023_4217_MOESM1_ESM.docx]

**Qing Liu et al. Mitochondrial genome of the diploid oat *Avena longiglumis*. Supplementary figures S1-S4.**

**Additional file 1: Figure S1.** Graphical assembly display of the mitogenome based on Unicycler using Illumina short reads and ONT long reads. **Figure S2.** The master circular mitogenome generated by manually merging the four contigs based on long repetitive sequences. **Figure S3.** Sequencing coverage based on Illumina short-reads. **Figure S4.** RNA-editing to create a stop codon of gene *ccmFC*.


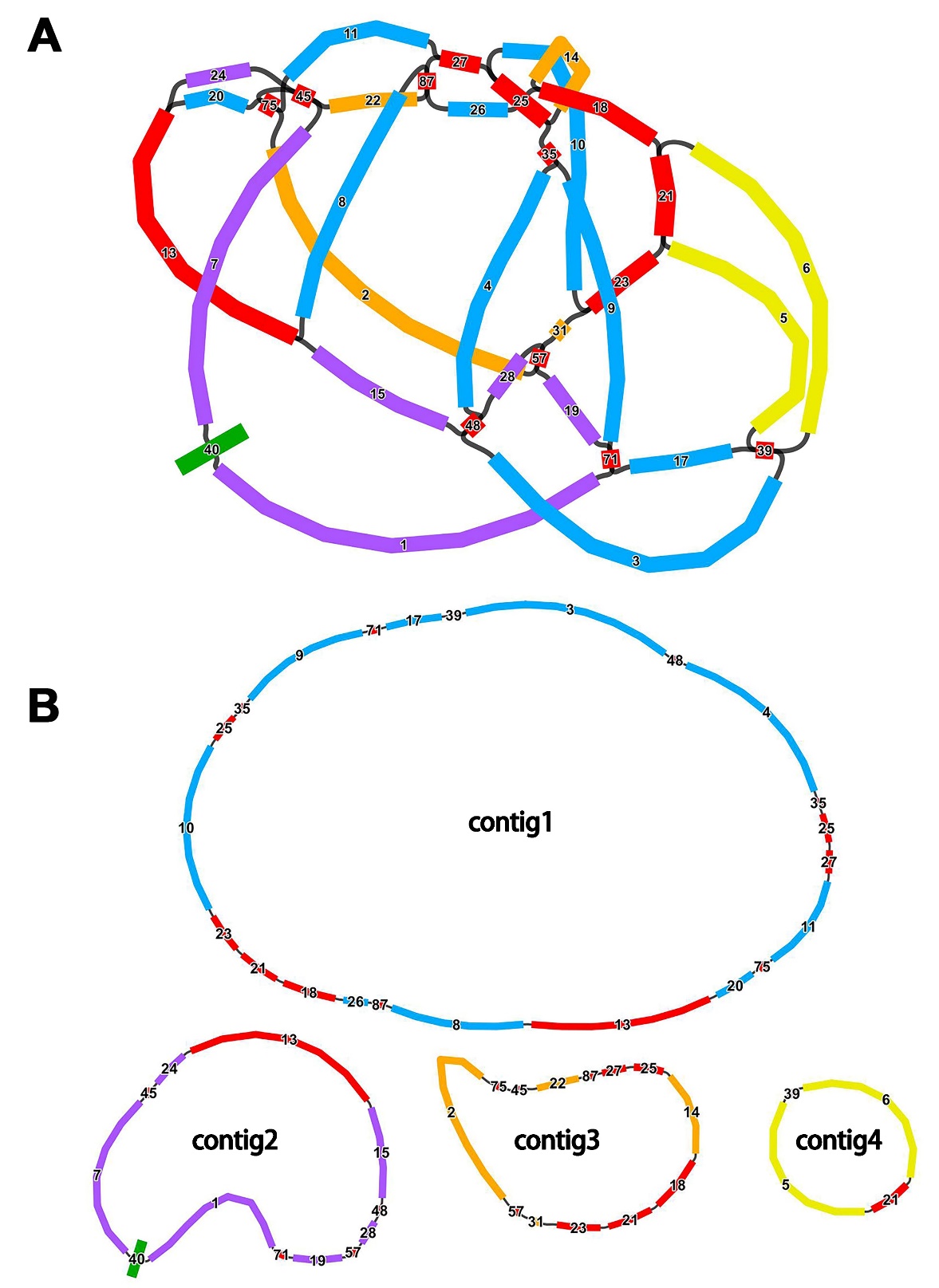


**Figure S1. Graphical assembly display of the mitogenome based on Unicycler using Illumina short reads and ONT long reads.** **A.** The 36 unitigs (high-confidence sub-contigs) showing several different tiling paths and overlapping regions. Each colored bar represents an assembled unitig numbered consecutively according to length (see Additional file 2: Table S2). **B.** Four circular contigs obtained by resolving duplicated regions based on Nanopore long-reads. Blue bars indicate unitigs of contig 1, purple of contig 2, orange of contig 3, and yellow of contig 4. Red bars are duplicated regions that occur on the same or different contigs. The green bar with high depth of coverage, has likely migrated from the plastome.

**Qing Liu et al. Mitochondrial genome of the diploid oat *Avena longiglumis*. Supplementary figures S1-S4.**


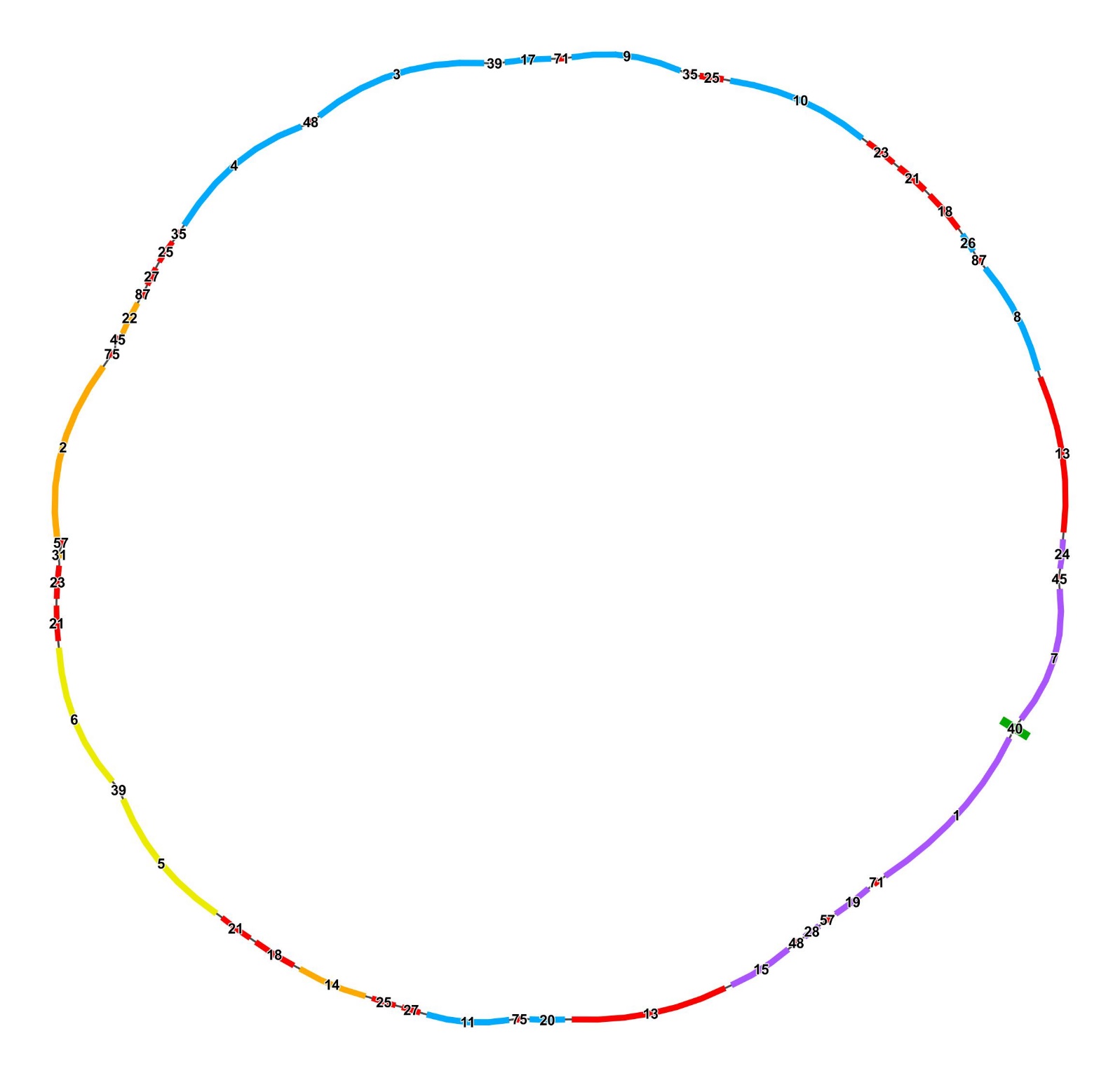


**Figure S2. The master circular mitogenome generated by manually merging the four contigs based on long repetitive DNA sequences.** Each node in Fig. S2 is corresponds to the node in Fig. S1. To present the mitochondrial genome as much as possible in the form of a master circle, we merged contig 1 and contig 2 based on repetitive unitig 13; we merged contig 1 and contig 3 based on repetitive unitig 23, 21 and 18; we merged contig 1 and contig 4 based on repetitive unitig 21.

**Qing Liu et al. Mitochondrial genome of the diploid oat *Avena longiglumis*. Supplementary figures S1-S4.**


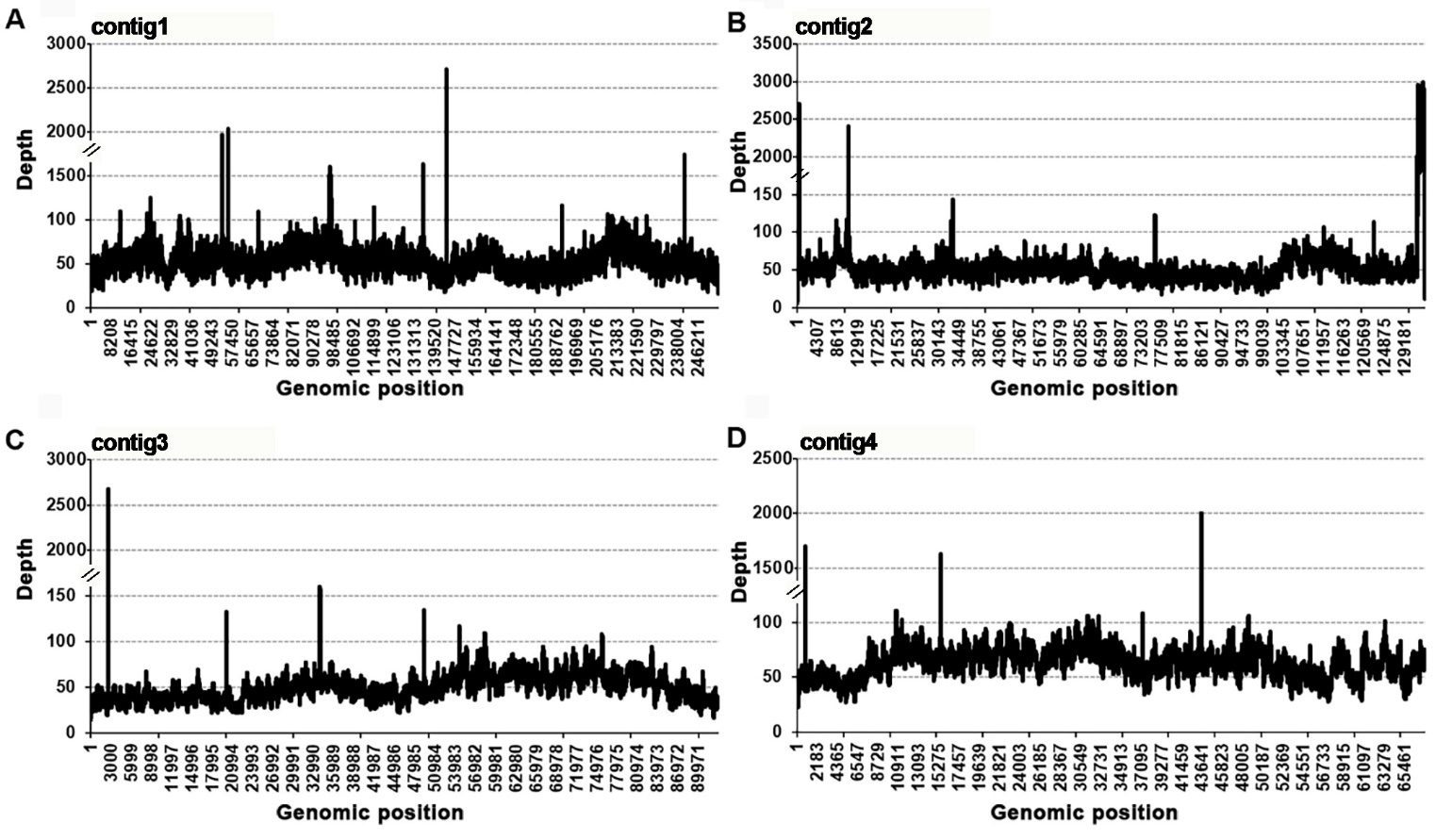


**Figure S3. Sequencing coverage based on Illumina short-reads. A-D.** Depth of coverage from contig 1 to contig 4, respectively. The abscissa indicates the genomic location within the contigs, and the ordinate indicates the sequencing depth. The average coverage of four contigs was about 56×.

**Qing Liu et al. Mitochondrial genome of the diploid oat *Avena longiglumis*. Supplementary figures S1-S4.**

**A**

**
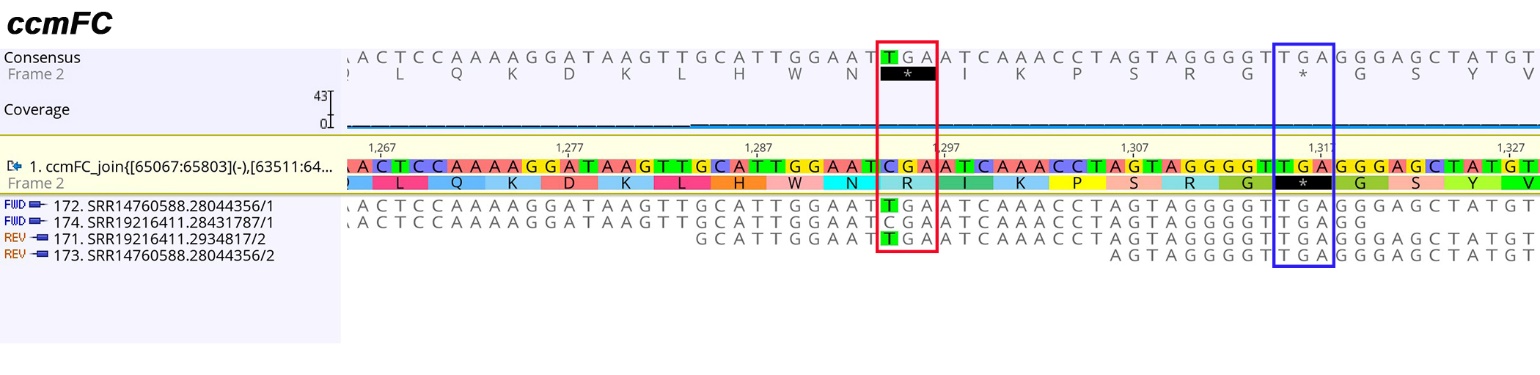
**

**B**


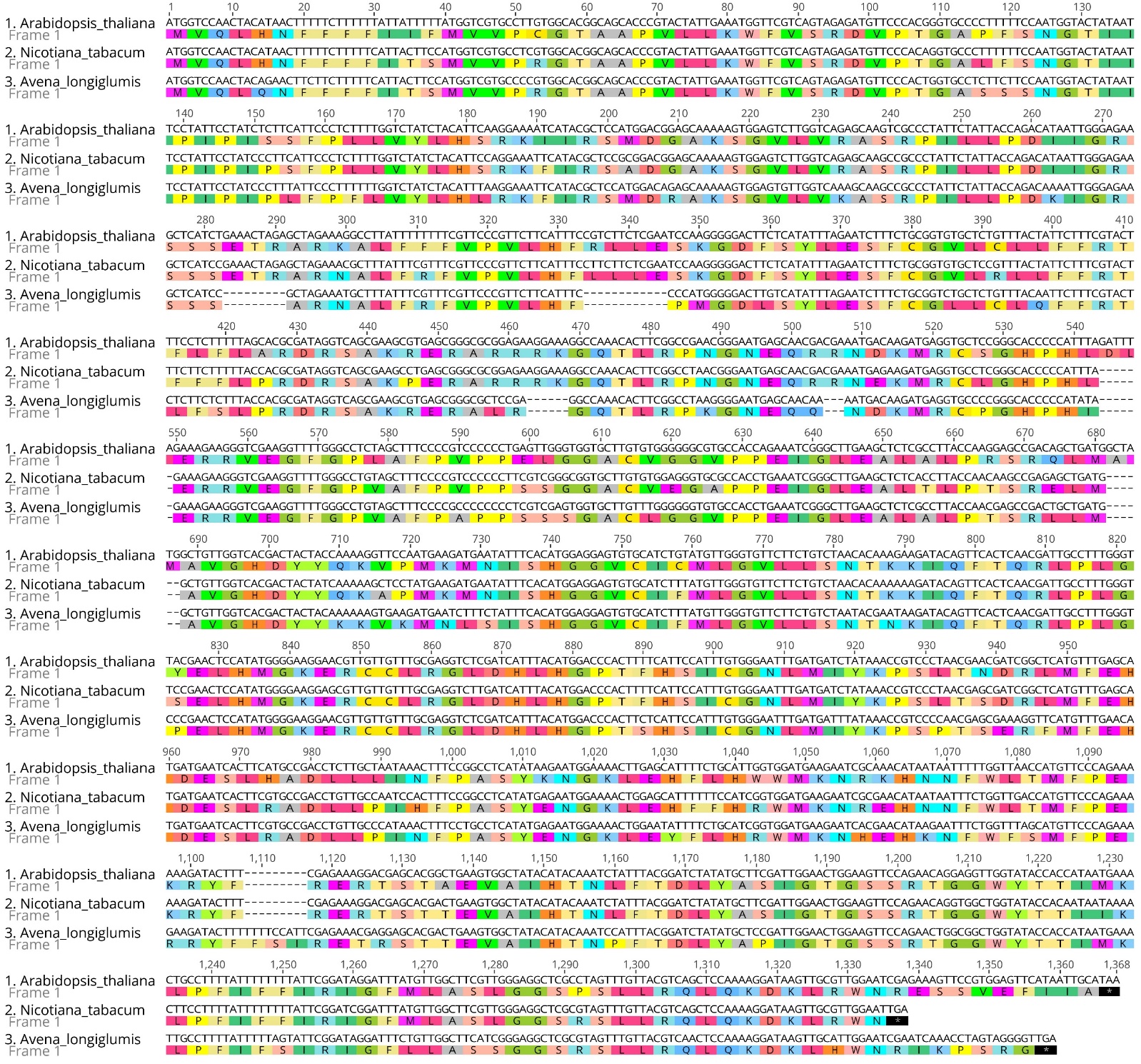


**Figure S4. RNA-editing to create a stop codon in the gene *ccmFC* of *Arabidopsis thaliana*, *Nicotiana tabacum* and *Avena longiglumis*. A. RNA editing create stop codon in *ccmFC* gene.** Though supported by relatively low coverage sequencing reads, we did find RNA editing events for the *ccmFC* gene to create stop codon. Blue box marks the stop codon on genomic DNA molecules and the red box marks the additional stop codon on edited RNA molecules. The highlighted green bases indicate the edited base C to T. **B. The alignment of the *ccmFC* gene.** These three *ccmFC* genes were extracted from: *Arabidopsis thaliana* (NC_037304: join{[189482:190261](+),[191220:191770(+)]}); *Nicotiana tabacum* (NC_006581: join{[307056:307823](+),[308777:309327(+)]}) and *Avena longiglumis* (OQ450323: join{[361936:362672](+),[363670:364228](+)}). In *N. tabacum*, the stop codon of *ccmFC* is TGA, and it has been noted in the *Arabidopsis thaliana*’s annotation that RNA editing events create stop codons for *ccmFC* genes. In this study, we also found that the stop codon of *ccmFC* genes in *Avena longiglumis* is also generated by RNA editing events as shown in Fig. S3A. These results shows that the stop codons can be created by RNA editing to maintain the conserved length of gene *ccmFC*.
